# Enhanced Terahertz Radiation Generation of Photoconductive Antennas Based on Manganese Ferrite Nanoparticles

**DOI:** 10.1038/srep46261

**Published:** 2017-04-10

**Authors:** Weien Lai, Oday Mazin Abdulmunem, Pablo del Pino, Beatriz Pelaz, Wolfgang J. Parak, Qian Zhang, Huaiwu Zhang

**Affiliations:** 1 Academy of Photoelectric Technology, HeFei University of Technology, HeFei, 230009, China; 2 Faculty of Physics, Philipps-Universität Marburg, Renthof 7, Marburg, 35032, Germany; 3 Centro Singular de Investigación en Química Biológica e Materiales Moleculares (CiQUS), and Departamento de Física de Partículas, Universidade de Santiago de Compostela, Santiago de Compostela, 15782, Spain; 4 Institute of Nano Biomedicine and Engineering, Key Laboratory for Thin Film and Microfabrication Technology of the Ministry of Education, Department of Instrument Science and Engineering, School of Electronic Information and Electrical Engineering, Shanghai Jiao Tong University, 800 Dongchuan RD, Shanghai, 200240, China; 5 State Key Laboratory of Electronic Films and Integrated Devices, University of Electronic Science and Technology of China, Chengdu, 610054, China

## Abstract

This paper presents a significant effect of manganese ferrite nanoparticles (MnFe_2_O_4_ NPs) on the increase of the surface photoconductivity of semiconductors. Herein, the optical characterization of photo-excited carriers of silicon coated with MnFe_2_O_4_ NPs was studied by using THz time-domain spectroscopy (THz-TDs). We observed that silicon coated with MnFe_2_O_4_ NPs provided a significantly enhanced attenuation of THz radiation in comparison with bare silicon substrates under laser irradiation. The experimental results were assessed in the context of a surface band structure model of semiconductors. In addition, photoconductive antennas coated with MnFe_2_O_4_ NPs significantly improved the efficiency of THz radiation generation and signal to noise ratio of the THz signal. This work demonstrates that coating with MnFe_2_O_4_ NPs could improve the overall performance of THz systems, and MnFe_2_O_4_ NPs could be further used for the implementation of novel optical devices.

Since nanoscale materials started to routinely appear in the literature almost two decades ago^1^, nanotechnology based on nanoparticles (NPs) has attracted interest by many researchers from very diverse areas, such as biology, physics, chemistry, engineering, materials science, and medicine. Recent developments based on nanomaterials have found a variety of uses in industry and life science, such as waste treatment, solar cells^2^, optoelectronics, light generation^3^, biomedicine^4^,^5^, *in-vitro* diagnostic reagents^6^, and biological labeling^7^, amongst others. These multifaceted applications mainly originate from specific size-related physicochemical properties present in many nanomaterials. When the size of bulk materials is reduced down to the nanoscale, the relative number of surface atoms to the total number of atoms in the material drastically increases^8^. Compared to bulk materials with ordered crystalline structure, the surface atoms of nanomaterials present weak and unsaturated bonding states, which drastically influence their surface properties. For instance, surface atoms of NPs present in general high chemical reactivity for catalytic processes. Besides the high surface-to-volume ratio which is present in all NPs, some NP materials also possess particular size-related properties when scaled down to the nanoscale. This is for example true for the case of localized surface plasmons (*e.g.*, plasmonic NPs^9^), fluorescence (*e.g.*, quantum dots, QDs^10^), superparamagnetism (*e.g.*, magnetic NPs^11^), photocatalysis (*e.g.*, titanium dioxide NPs^12^), *etc.*

Recently, nanotechnology has extended into the field of terahertz (THz) technology. THz technology has become increasingly attractive because of its potential applications^13^,^14^,^15^,^16^. For the application of THz technology, a THz system requires two main components: an emitter and a detector^16^,^17^,^18^,^19^. THz emitters can be produced by various techniques, such as photoconductive (PC) antennas^17^,^18^,^20^, superconductor^21^ and nonlinear crystals^22^. However, in comparison with other techniques^21^,^22^,^23^,^24^, PC antennas are key players due to their high efficiency. In general, PC antennas are fabricated on semiconductor substrates, such as low-temperature-grown GaAs^19^,^25^. In order to improve their performance, a significant body of work has been focused on optimizing the structures and substrates for PC antennas^26^,^27^,^28^. However, most of these PC antennas have complex structures, which rely on sophisticated fabrication techniques. Therefore, one key scientific challenge is to improve the performance of PC antennas by cost-effective simple methods^26^,^29^,^30^,^31^,^32^. In this work, the effect of coating a silicon substrate with MnFe_2_O_4_ NPs on its PC properties was investigated. Based on this an approach to improve the performance of PC antennas by utilizing a coating based on MnFe_2_O_4_ NPs is presented. MnFe_2_O_4_ NPs are low-cost materials, present high-stability and environmental compatibility, which makes this type of coatings a potential candidate for industrial applications.

## Results and Discussion

### Synthesis and characterization of manganese ferrite nanoparticles (MnFe_2_O_4_ NPs)

**Figure 1:**
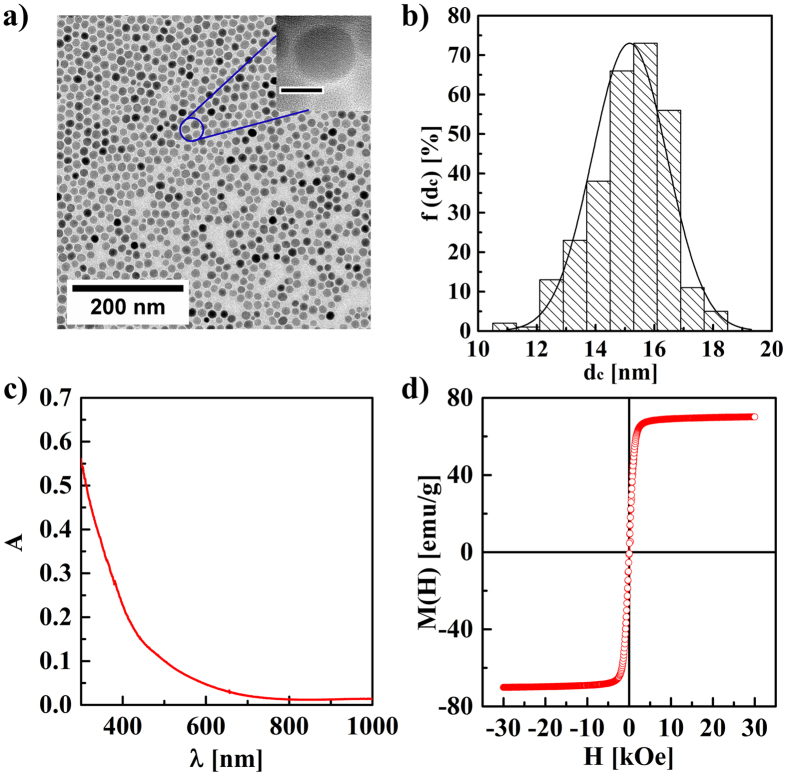
(**a**) TEM bright field image of MnFe_2_O_4_ NPs (scale bar: 200 nm). The insert shows a HRTEM image of one individual NP (scale bar: 15 nm). (**b**) The corresponding histogram for the distribution frequency of the core diameter f(d_c_), as produced by analysis of the TEM image with the free software ImageJ is based on analyzing >300 NPs. The mean diameter of the inorganic cores was determined to be d_c_ = 15.06 ± 1.2 nm. (**c**) UV-Vis absorption spectra A(λ) of MnFe_2_O_4_ NPs in chloroform. (**d**) Magnetization versus magnetic field M(H) curve of MnFe_2_O_4_ NPs, measured at 300 K using a SQUID magnetometer. The saturation magnetization was found to be about M_s_ = 72 emu/g.

Monodisperse MnFe_2_O_4_ NPs were synthesized *via* thermal decomposition as described in the Methods Section, based on a previously published protocol^11^. The resulting MnFe_2_O_4_ NPs were well dispersed in organic solvents, having long-time colloidal stability. The elemental components in the MnFe_2_O_4_ NPs were determined by inductively coupled plasma mass spectrometry (ICP-MS), confirming that the Mn:Fe ratio was approximately 1:2 (*cf.* Table S1 in the Supporting Information).The transmission electron microscopy (TEM) and high-resolution TEM images (HRTEM) in Fig. 1a demonstrate that the MnFe_2_O_4_ NPs present an uniform size and spherical structure. The size distribution of the NPs was analyzed by using the software ImageJ, yielding a diameter of inorganic core (d_c_) of 15 nm with variation σ < 5% (Fig. 1b). The hydrodynamic diameter of the MnFe_2_O_4_ NPs dispersed in chloroform was measured by dynamic light scattering (DLS), indicating a narrow size distribution (*cf.* Figure S1 in the Supporting Information). The absorption spectrum of the MnFe_2_O_4_ NPs was measured by UV-Vis absorption spectrophotometry (Fig. 1c). The spectra indicate that the NPs have no specific absorption in the NIR spectral region. The magnetic properties of the MnFe_2_O_4_ NPs were measured by a superconducting quantum interference device (SQUID), and the resulting magnetization *versus* magnetic field curve M(H) is shown in Fig. 1d. This curve exhibits a smooth loop without hysteresis loss, with a saturated magnetization value (M_s_) of 72 emu/g. Thus, the MnFe_2_O_4_ NPs exhibit excellent superparamagnetic behavior.

### Optical characterization of MnFe_2_O_4_ NPs on silicon (NPOS) under laser irradiation

**Figure 2:**
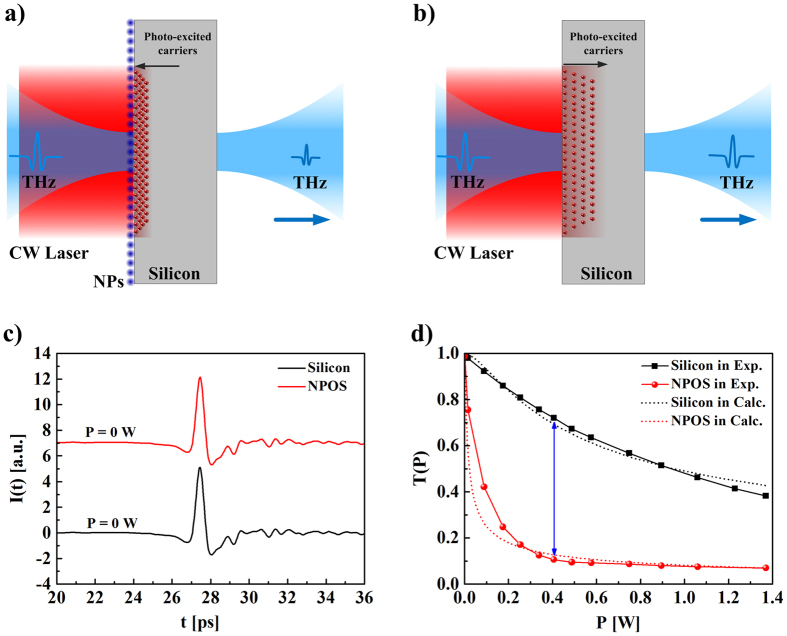
Schematic representation of a THz pulse transmitted through a NPOS substrate (**a**) and a bare silicon substrate (**b**) under CW laser irradiation. (**c**) Waveforms of THz pulses I(t) transmitted through silicon and NPOS substrates. (**d**) Amplitude transmissions T(P) of the THz pulses transmitted through the silicon and NPOS substrates under laser irradiation with different lased powers P. The dotted lines represent fits to the experimental data.

NPOS substrates were fabricated by spin-coating of the MnFe_2_O_4_ NPs on high resistivity silicon as substrate (details are described in Methods Section, SEM images are shown in Figure S2). A bare silicon substrate without NPs was used as control. To study the optical properties of NPOS substrate under laser irradiation (continuous wave (CW) excitation at 808 nm), THz time-domain spectroscopy (THz-TDS) in transmission mode was simultaneously performed, as shown in Fig. 2. The spot diameter of the CW laser was about 4 mm, fully overlapping with the THz beam on the surface of the samples. The THz beam was directed to the sample at normal incidence by a focusing lens, as shown in Fig. 2. The frequency bandwidth in the THz system was limited to 2.5 THz, since outside this frequency range the signal was too weak to obtain an acceptable signal-to-noise ratio. The optical properties of the NPOS substrate were investigated by changing the irradiation power of the CW laser. In addition, in order to understand the THz response of NPs under laser irradiation, a bare quartz substrate and a NPs-coated quartz substrate were used as control group.

**Figure 3:**
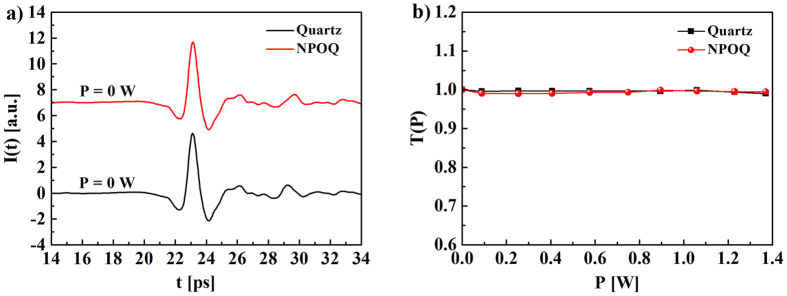
(**a**) Waveforms of THz pulses transmitted through quartz and MnFe_2_O_4_ NPs on quartz (NPOQ) substrates. (**b**) Amplitude of the transmissions of the THz pulses transmitted through quartz and NPOQ substrates under laser irradiation with different laser powers, demonstrating that the NPs do not absorb THz excitation.

For THz transmission measurements under laser irradiation, the THz signals transmitted through the samples consisted of pulsed waveforms. The waveforms of THz pulses transmitted through the silicon and NPOS substrates under the laser irradiation with different powers are shown in Figure S9 (*cf.* Figure S9 in the Supporting Information). The amplitudes of the THz pulses transmitted through the NPOS substrates, as well as the amplitudes of the THz pulses transmitted through bare silicon substrates, decreased as expected upon increasing the power of the laser irradiation. Furthermore, under no laser irradiation, the waveform of THz pulse transmitted through the NPOS substrate was almost identical to the one of the bare silicon substrate, as shown in Fig. 2. This demonstrates that the absorption of MnFe_2_O_4_ NPs in the THz frequency range is negligible. Moreover, under no laser irradiation (P = 0), the waveform of THz pulses transmitted through NPOQ substrates was almost identical to the one through bare quartz substrates, as shown in Fig. 3a. The coefficients of the transmissions of THz pulses transmitted through NPOQ substrates, as well as the one transmitted through bare quartz substrates, was almost identical and constant (*T* ≈ 1) upon increasing the power of the laser irradiation, as shown in Fig. 3b. This demonstrates that the absorption of MnFe_2_O_4_ NPs at 808 nm wavelength of CW laser excitation is negligible.

To account for the differences of THz transmissions between NPOS and bare silicon substrates under laser irradiation, we further compared the amplitudes of the THz pulses transmitted through NPOS substrates with the ones transmitted through bare silicon substrates. The amplitudes of both pulses were normalized with respect to the amplitude of the THz pulse transmitted through bare silicon under no laser irradiation. We found that in both cases, the amplitudes of the transmissions slowly decreased by increasing the power of the laser irradiation, as shown in Fig. 2d. The transmission-dropping in the case of the NPOS substrate was significantly higher than in the bare silicon substrate. When the power of laser irradiation was about P ≈ 0.41 W, the transmission change (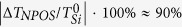) in NPOS substrate was significantly higher than the transmission change (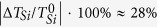) of the bare silicon substrate, as shown in Fig. 2d. On the other hand, the amplitude transmissions through NPOS were in a good agreement with that predicted by simulations obtained by a theoretical model based on band theory, which is described in the Supporting Information. The proposed modeling and the experiments on bare silicon substrates yielded similar results, except for low irradiation powers (P < 0.2 W). We speculate that this discrepancy may arise from partial beam blocking by the NPOS substrates, resulting in a less efficient production of photo-excited carriers. Yet for laser irradiation above P = 0.2 W, the effect of the aforementioned phenomenon is negligible. Since the amplitude of the THz pulse transmitted through a sample is inherently correlated with the conductivity of the conducting layer of the sample, for the same irradiation power, the conductivity in a NPOS substrate is significantly higher than in a bare silicon substrate. This explains the higher density of photo-excited carriers in NPOS as compared to bare silicon substrates. In parallel, we also investigated THz transmission amplitudes through different NPOS substrates based on a series of different NPs under laser irradiation with different powers P (*cf.* Figures S3, S4 and S5 in the Supporting Information), and we compared different magnetic NPs with similar size to exclude the effect of magnetic response of NPs on the photoconductivity of the semiconductors (*cf.* Figures S7 and S8 in the Supporting Information). We found that all NP-coated silicon substrates slightly decreased the transmissions as compared to bare silicon substrates with increasing the laser power. However, the NPOS substrates based on MnFe_2_O_4_ NPs exhibited the biggest effect of all tested NPs, which could be explained by the properties of materials tested here in the Supporting Information. Hence, our experiments demonstrate that coating with MnFe_2_O_4_ NPs significantly provides an enhanced attenuation of THz radiation in comparison with bare silicon substrates under laser irradiation.

### Performance of MnFe_2_O_4_ NP coated PC antennas

**Figure 4:**
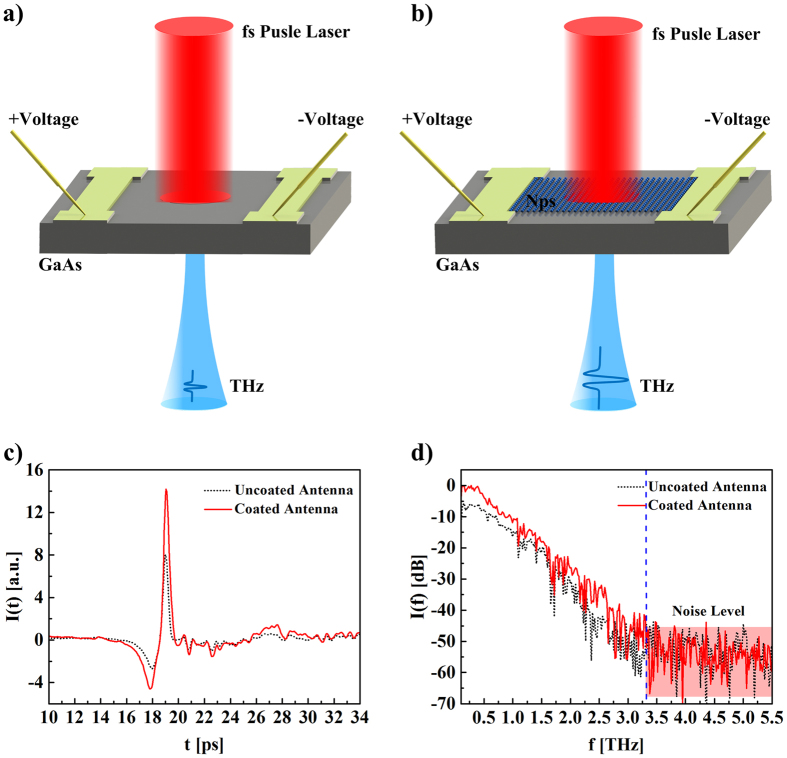
Experimental schematic of THz pulses generated from (**a**) an uncoated PC antenna and (**b**) from a PC antenna coated with MnFe_2_O_4_ NPs under fs laser pumping. (**c**) Waveforms of THz pulses generated from the uncoated PC antenna and the PC antenna coated with MnFe_2_O_4_ NPs. (**d**) The emission spectra of THz pulses generated from the uncoated PC antenna and the PC antenna coated with MnFe_2_O_4_ NPs.

PC antennas were used to verify the potential application of MnFe_2_O_4_ NPs in the THz region. The PC antenna used herein consisted of a simple stripline structure of metal film and a low-temperature-grown GaAs substrate. A schematic of the PC antenna used herein is shown in Fig. 4. A femtosecond (fs) pulsed laser (central wavelength of 780 nm, 155 fs pulse duration) was used to pump the PC antenna to generate THz radiation. The PC antenna was biased with an AC voltage of frequency. First, the fs laser was focused on the uncoated PC antenna to generate THz radiation as reference signal. Equivalently, the NPs-coated antenna was used to generate THz radiation as sample signal. These measurements were performed 6 times, to ensure the reproducibility of the experimental results.

From this experiment, it is observable that, under equal pumping conditions, the amplitude of the THz signal from the NP-coated PC antenna (sample signal) was significantly higher than that of the reference signal (uncoated antenna), as illustrated in Fig. 4c. Likewise, in the time-frequency transformation, the signal-to-noise ratio of the NP-coated antenna was improved as compared to the uncoated antenna, as shown in Fig. 4d. The electric field 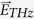 of THz radiation as generated from a PC antenna^14^,^25^,^29^ is expressed as 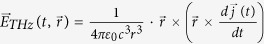. Here, 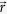 is the vector in the direction of the observation and 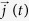 is the time-dependent photocurrent density. According to this equation, the correlation between the electric field of THz radiation and the photocurrent density can be described by 
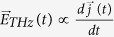
14. Therefore, the electric field of THz radiation is enhanced by increasing the photocurrent density in the PC antenna. The coating of MnFe_2_O_4_ NPs on the PC antenna can increase the carrier density, so that the photocurrent density also increases in the PC antenna. The experimental results of the here used PC antennas demonstrate that addition of MnFe_2_O_4_ NPs can increase the THz radiation generation efficiency of PC antennas. In addition, the MnFe_2_O_4_ NPs improve the signal-to-noise ratio of the THz signal generated from the PC antenna, which plays an important role in the performance of THz systems. Therefore, the usage of MnFe_2_O_4_ NPOS substrates can improve the performance of THz systems.

## Conclusion

In conclusion, we demonstrated that coating with MnFe_2_O_4_ NPs can be used to improve the performance of PC antennas in the THz region. Our experiments demonstrate that coatings with MnFe_2_O_4_ NPs provided a new approach to increase the photocurrent density on silicon under CW illumination. In order to understand the effect of MnFe_2_O_4_ NPs on photo-excited silicon, a semiconductor model was proposed to describe this phenomenon. We used this model to calculate the transmission amplitudes of THz pulses transmitted through NPOS and bare silicon substrates under laser irradiation with different powers. Because the effect of MnFe_2_O_4_ NPs on silicon significantly provides an enhanced attenuation of terahertz wave, NPOS has the potential to be used as an optical modulator in the THz region. This may lead to a cost-efficient component for THz-TDS systems operating in transmission mode. Hence, MnFe_2_O_4_ NPs have the potential application to increase the overall performance of THz-TDS systems. Furthermore, MnFe_2_O_4_ NPs could be used for the implementation of novel optical devices.

## Methods

### Synthesis of monodisperse MnFe_2_O_4_ NPs

The synthesis method for monodisperse MnFe_2_O_4_ NPs (core diameter ~15 nm) is based on the previously published protocols from Sun and Zhang^11^,^33^. In order to acquire the desired size, the synthesis was separated into a seed synthesis process and a seed-mediated growth process.

Briefly, iron(III) acetylacetonate (Fe(acac)_3,_ 2 mmol), manganese(II) acetylacetonate (Mn(acac)_2_, 1 mmol) and 1,2-hexadecanediol (10 mmol) were placed in a 100 mL three-neck flask in the presence of oleic acid (OLA, 6 mmol), oleylamine (OLAM, 6 mmol), and 10 mL of benzyl ether. Under magnetic stirring, the mixture was firstly degassed at 100 °C with vacuum for 30 min. Under the protection of nitrogen flow, the mixture was slowly heated up to 200 °C and aged for 2 h, and then heated again to 300 °C with keeping this temperature for 1 h. The reaction was stopped by removing the heating mantle, followed by the addition of ethanol (~40 mL) to precipitate black-colored seeds with centrifugation (3000 rpm, 10 min). After removing the supernatant, the precipitate was dissolved in hexane in the presence of 10 μL mixture of OLA and OLAM, which served for stabilizing the NPs. Centrifugation (3000 rpm, 10 min) was applied again in order to remove aggregates. The product, *i.e.* the supernatant with MnFe_2_O_4_ NPs of around 10 nm core diameter, was harvested by centrifugation (3000 rpm, 10 min) under the addition of sufficient ethanol. The NP precipitate was then redispersed in chloroform at a concentration of 20 mg/mL.

In order to produce MnFe_2_O_4_ NPs of 15 nm core diameter, a seed-mediated growth process was used by growing a shell on the previously described 10 nm diameter MnFe_2_O_4_ NPs seeds. Specifically, 2 mmol of Fe(acac)_3_, 1 mmol of Mn(acac)_2_, 10 mmol of 1,2-hexadecanediol were mixed with 50 mg MnFe_2_O_4_ seeds (dissolved in 2.5 mL CHCl_3_), 2 mmol of OLA, 2 mmol of OLAM and 20 mL of benzyl ether. The mixture was magnetically stirred at 100 °C for 20 min under vacuum to remove CHCl_3_. Under the protection of nitrogen flow, the temperature was raised slowly to 200 °C, and the solution was left for 1 h at this temperature. Then the temperature was again increased with the same heating speed up to reflux (~300 °C) for 30 min. The black-colored mixture was cooled down to room temperature (R.T.) by removing the heating mantle. The NPs were washed by repeated precipitation and redissolution as described above. Finally, the resulting 15 nm diameter MnFe_2_O_4_ NPs were dissolved in 20 mL of CHCl_3_.

### Fabrication of manganese ferrite nanoparticles on a silicon (NPOS)

The monodisperse manganese ferrite NPs were immobilized on a silicon substrate to fabricate a NPOS system *via* a spin coating process^34^. Typically, a silicon substrate (resistivity >2000 Ω · cm, cut from a 0.5-mm thick wafer), was firstly cleaned 3 times with acetone solution. Then 50 μL of manganese ferrite NPs (dispersed in chloroform at a NP concentration of 2 mg/mL) was casted on the surface of the silicon substrate with a rotating speed of 100 rpm for 10 s.

## Additional Information

**How to cite this article:** Lai, W. *et al*. Enhanced Terahertz Radiation Generation of Photoconductive Antennas Based on Manganese Ferrite Nanoparticles. *Sci. Rep.* **7**, 46261; doi: 10.1038/srep46261 (2017).

**Publisher's note:** Springer Nature remains neutral with regard to jurisdictional claims in published maps and institutional affiliations.

## Supplementary Material

Supplementary Information

## References

[b1] Chatelain A., Bonard J. M., Buttet J. & Monot R. Small particles and inorganic clusters. Preface. Eur Phys J D 9, U1–U1 (1999).

[b2] Lee J., Mahendra S. & Alvarez P. J. J. Nanomaterials in the Construction Industry: A Review of Their Applications and Environmental Health and Safety Considerations. Acs Nano 4, 3580–3590 (2010). 20695513 10.1021/nn100866w

[b3] Rizzo A. . Blue light emitting diodes based on fluorescent CdSe/ZnS nanocrystals. Appl Phys Lett 90, 051106 (2007).

[b4] Daniel M. C. & Astruc D. Gold nanoparticles: Assembly, supramolecular chemistry, quantum-size-related properties, and applications toward biology, catalysis, and nanotechnology. Chem Rev 104, 293–346 (2004). 14719978 10.1021/cr030698+

[b5] Ashraf S. . Gold-Based Nanomaterials for Applications in Nanomedicine. Top Curr Chem 370, 169–202 (2016). 26589509 10.1007/978-3-319-22942-3_6

[b6] Azzazy H. M. E. & Mansour M. M. H. *In vitro* diagnostic prospects of nanoparticles. Clin Chim Acta 403, 1–8 (2009). 19361470 10.1016/j.cca.2009.01.016

[b7] Zhang C. L. . Gold Nanoclusters-Based Nanoprobes for Simultaneous Fluorescence Imaging and Targeted Photodynamic Therapy with Superior Penetration and Retention Behavior in Tumors. Advanced Functional Materials 25, 1314–1325 (2015).

[b8] Goesmann H. & Feldmann C. Nanoparticulate Functional Materials. Angewandte Chemie, International Edition 49, 1362–1395 (2010). 20108287 10.1002/anie.200903053

[b9] Petryayeva E. & Krull U. J. Localized surface plasmon resonance: Nanostructures, bioassays and biosensing-A review. Anal Chim Acta 706, 8–24 (2011). 21995909 10.1016/j.aca.2011.08.020

[b10] Khalid W. . Immobilization of Quantum Dots via Conjugated Self-Assembled Monolayers and Their Application as a Light-Controlled Sensor for the Detection of Hydrogen Peroxide. ACS Nano 5, 9870–9876 (2011). 22070721 10.1021/nn2035582

[b11] Zhang Q. . Model Driven Optimization of Magnetic Anisotropy of Exchange-Coupled Core-Shell Ferrite Nanoparticles for Maximal Hysteretic Loss. Chem Mater 27, 7380–7387 (2015). 10.1021/acs.chemmater.5b03261 PMC6519962 31105383

[b12] Pelaez M. . A review on the visible light active titanium dioxide photocatalysts for environmental applications. Appl Catal B-Environ 125, 331–349 (2012).

[b13] Horiuchi N. View From … Teranano 2011 Terahertz Nano-Exploration. Nat Photonics 6, 82–83 (2012).

[b14] Jepsen P. U., Cooke D. G. & Koch M. Terahertz spectroscopy and imaging - Modern techniques and applications. Laser Photonics Rev 5, 124–166 (2011).

[b15] Ferguson B. & Zhang X. C. Materials for terahertz science and technology. Nature Materials 1, 26–33 (2002). 12618844 10.1038/nmat708

[b16] Lai W. E., Zhang H. W., Zhu Y. H. & Wen Q. Y. A Novel Method of Terahertz Spectroscopy and Imaging in Reflection Geometry. Appl Spectrosc 67, 36–39 (2013). 23317668 10.1366/12-06713

[b17] Cai Y. . Design and performance of singular electric field terahertz photoconducting antennas. Appl Phys Lett 71, 2076–2078 (1997).

[b18] Jepsen P. U., Jacobsen R. H. & Keiding S. R. Generation and detection of terahertz pulses from biased semiconductor antennas. Journal of the Optical Society of America B 13, 2424–2436 (1996).

[b19] Zhang J., Hong Y., Braunstein S. L. & Shore K. A. Terahertz pulse generation and detection with LT-GaAs photoconductive antenna. IEE Proceedings - Optoelectronics 151, 98–101 (2004).

[b20] Castro-Camus E., Lloyd-Hughes J. & Johnston M. B. Three-dimensional carrier-dynamics simulation of terahertz emission from photoconductive switches. Phys Rev B 71 (2005).

[b21] Kashiwagi T. . Generation of electromagnetic waves from 0.3 to 1.6 terahertz with a high-Tc superconducting Bi_2_Sr_2_CaCu_2_O_8_+ δ intrinsic Josephson junction emitter. Appl Phys Lett 106, 2394 (2015).

[b22] Carey J. J. . Terahertz pulse generation in an organic crystal by optical rectification and resonant excitation of molecular charge transfer. Appl Phys Lett 81, 4335–4337 (2002).

[b23] Zhong H., Karpowicz N. & Zhang X. C. Terahertz emission profile from laser-induced air plasma. Appl Phys Lett 88 (2006).

[b24] Kadowaki K. . Quantum terahertz electronics (QTE) using coherent radiation from high temperature superconducting Bi_2_Sr_2_CaCu_2_O_8_+ delta intrinsic Josephson junctions. Physica C 491, 2–6 (2013).

[b25] Awad M., Nagel M., Kurz H., Herfort J. & Ploog K. Characterization of low temperature GaAs antenna array terahertz emitters. Appl Phys Lett 91 (2007).

[b26] Han S. P. . Compact fiber-pigtailed InGaAs photoconductive antenna module for terahertz-wave generation and detection. Opt Express 20, 18432–18439 (2012). 23038394 10.1364/OE.20.018432

[b27] Mittendorff M. . Large area photoconductive terahertz emitter for 1.55 mu m excitation based on an InGaAs heterostructure. Nanotechnology 24 (2013). 10.1088/0957-4484/24/21/214007 23619031

[b28] Tanoto H. . Nano-antenna in a photoconductive photomixer for highly efficient continuous wave terahertz emission. Scientific Reports 3 (2013). 10.1038/srep02824 PMC3792413 24100840

[b29] Gao Y. H. . Analysis of terahertz generation via nanostructure enhanced plasmonic excitations. Journal of Applied Physics 106 (2009).

[b30] Tong J. Y., Muthee M., Chen S. Y., Yngvesson S. K. & Yan J. Antenna Enhanced Graphene THz Emitter and Detector. Nano Letters 15, 5295–5301 (2015). 26218887 10.1021/acs.nanolett.5b01635

[b31] Young C. D. Exploring terahertz pulse enhancement through gold nanoparticle deposition. Dissertations & Theses - Gradworks (2009).

[b32] Park S. G., Choi Y., Oh Y. J. & Jeong K. H. Terahertz photoconductive antenna with metal nanoislands. Opt Express 20, 25530–25535 (2012). 23187370 10.1364/OE.20.025530

[b33] Sun S. . Monodisperse MFe_2_O_4_ (M = Fe, Co, Mn) Nanoparticles. J. Am. Chem. Soc. 126, 273–279 (2004). 14709092 10.1021/ja0380852

[b34] Sabir N. . Photo-electrochemical Bioanalysis of Guanosine Monophosphate Using Coupled Enzymatic Reactions at a CdS/ZnS Quantum Dot Electrode. Small 11, 5844–5850 (2015). 26395754 10.1002/smll.201501883

