# Supplementary material for: Enhanced Terahertz Radiation Generation of Photoconductive Antennas Based on Manganese Ferrite Nanoparticles
Source: Sci Rep. 2017 Apr 10;7:46261. doi: 10.1038/srep46261 (PMC5385867; doi:10.1038/srep46261)
Supplement: Supplementary Information [file srep46261-s1.pdf]

Supporting Information

Enhanced Terahertz Radiation Generation of Photoconductive Antennas Based on Manganese Ferrite Nanoparticles

Weien Lai, Oday Mazin Abdulmunem, Pablo del Pino, Beatriz Pelaz, Wolfgang J. Parak, Qian Zhang and Huaiwu Zhang

1. Dynamic Light Scattering (DLS) measurements

The average size distribution of $MnFe_2O_4$ NPs in chloroform was determined by DLS (shown in Figure S1). The measurements were repeated three times. The mean diameter d_h in chloroform as derived from the number distribution was determined to be 14.8 nm. This demonstrates that the NPs were well dispersed in organic solvent without aggregation.

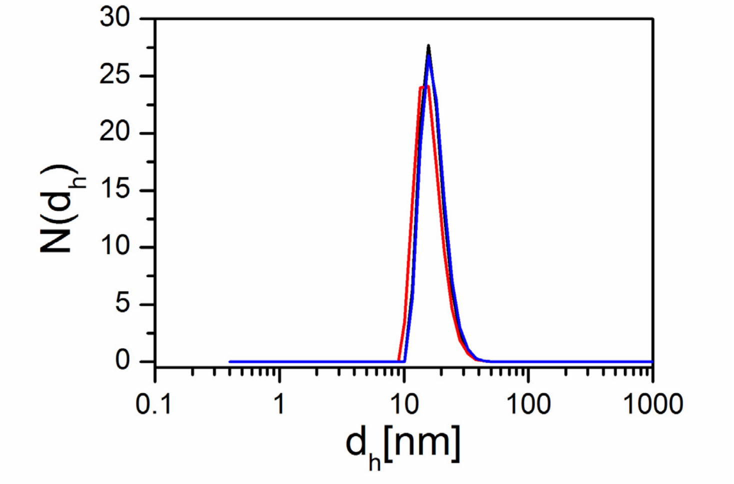

Figure S1. Number distribution $N(d_h)$ of the solution-based diameter of $MnFe_2O_4$ NPs dispersed in chloroform. The mean diameter is about 14.8 nm.

2. Inductively coupled plasma mass spectrometry (ICP-MS) measurement

The elemental composition of the $MnFe_2O_4$ NPs and the NP concentration in solution were measured by ICP-MS. The NP sample was prepared with the following protocol for ICP-MS measurements. Briefly, 10 µL of $MnFe_2O_4$ NPs in organic solvent was added into a tube, the solvent was evaporated, and 400 µL of fresh prepared aqua regia (HCl/ HNO_3 =3:1 (v/v)) was injected for the digestion. During this time, the NP dissolved into small fragments. After 4 hours digestion, 9.6 mL of 2 % HCl solution was added to the tube. Addition of

the acids resulted in a 1000 times total dilution. Finally, the diluted solution was injected into the ICP-MS equipment (Agilent 7700) for measuring the content of manganese and iron. The result of the element concentration was provided in ppb (parts per billion, 1 ppb corresponds to 1 μg/L).

Table S1. The elemental composition of the NPs as analyzed by ICP-MS. $C_{(Fe)}$ and $C_{(Mn)}$ refer to the mass concentrations of Fe and Mn, respectively.

**Sample material** (in $CHCl_3$)	**Fe** (mg/mL)	**Mn** (mg/mL)	**Total** (mg/mL)	$C_{(Fe)}$: $C_{(Mn)}$
$MnFe_2O_4$ NPs	3.56	1.65	7.25	2.12

3. Characterization of $MnFe_2O_4$ NPs on the silicon

Manganese ferrite nanoparticles on the silicon (NPOS) were characterized by scanning electron microscopy (SEM, FEI-Sirion 200). The $MnFe_2O_4$ NPs layer was obvious, as shown in Figure S2.

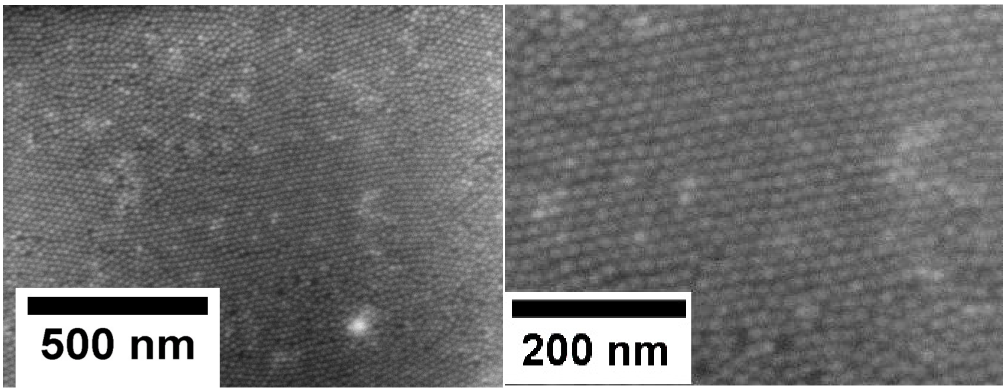

Figure S2. SEM images of $MnFe_2O_4$ NPs on the substrate.

4. Amplitude transmissions measurements based on different types of NPs

In this experiment, a series of NPs of materials others than $MnFe_2O_4$ were studied as comparison. Herein, we investigated four different types of NPs as control group to confirm the importance of the combination of

elements concerning the modification of THz antennas. The control samples were silver nanoparticles (Ag NPs, $d_c \approx 12$ nm), iron oxide nanoparticles (Fe_3O_4 NPs, $d_c \approx 27$ nm), titanium dioxide nanoparticles (TiO_2 NPs, $d_c \approx$ 11 nm), and gold nanoparticles (Au NPs, $d_c \approx 16$ nm), respectively. The core diameters d_c of all samples were determined by transmission electron microscopy (TEM).

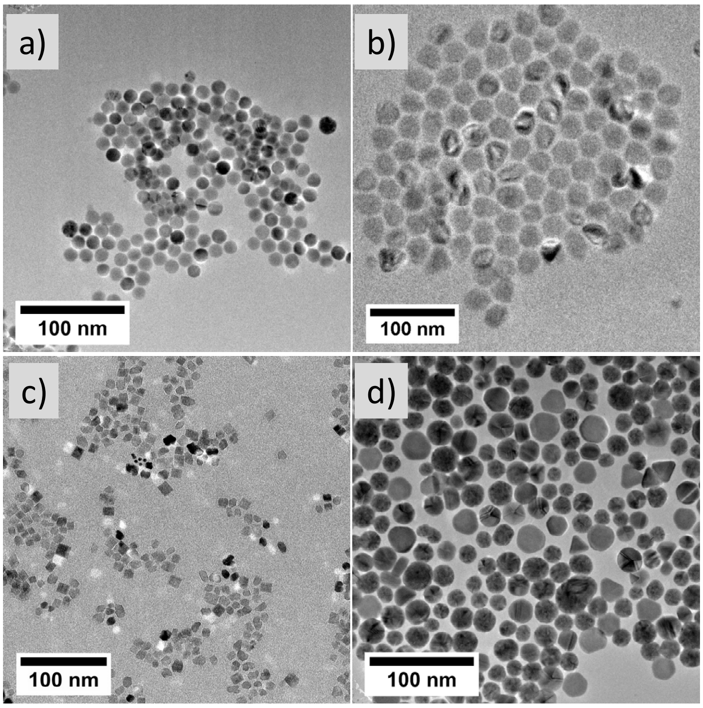

Figure S3. TEM images of four different types of NPs. a) Ag NPs; b) Fe_3O_4 NPs; c) TiO_2 NPs and d) Au NPs. All the samples were dispersed in chloroform before the actual TEM measurements carried out in vacuum. The scale bars indicate 100 nm.

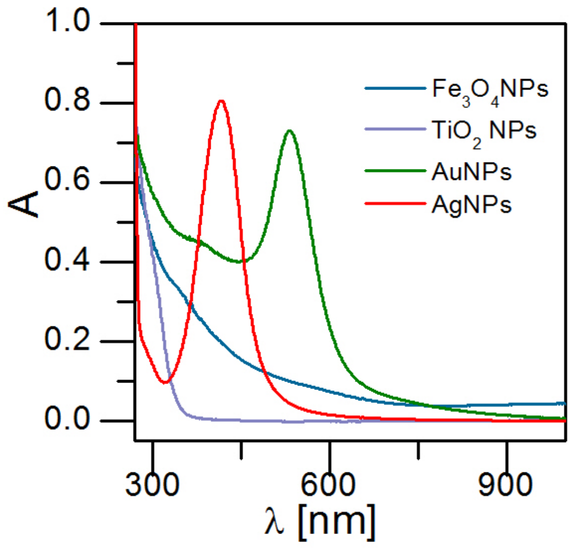

Figure S4. UV-Vis absorption spectra of four different types of NPs dispersed in chloroform: Fe_3O_4 NPs, TiO_2 NPs, Au NPs, and Ag NPs. From the spectra one can see that none of the samples has specific absorption at 808 nm, which means that there is no signal interference with the terahertz amplitude transmission experiments.

With the same fabrication process as used for the $MnFe_2O_4$ NPs, the four different types of NPs were immobilized with same molar concentration on silicon substrates in order to achieve Ag NPs on silicon, Fe_3O_4 NPs on silicon, TiO_2 NPs on silicon, and Au NPs on silicon, respectively. These four substrates immobilized with the different types of nanomaterials were tested concerning the transmissions of THz pulses. As a result transmission was found to be almost identical and constant ($T \approx 1$) with increasing the power of laser irradiation, similar as recorded for $MnFe_2O_4$ NPs on silicon. Afterwards, under laser irradiation with different powers, THz amplitude transmissions through silicon coated with the five different types of NPs (Ag NPs, Fe_3O_4 NPs, TiO_2 NPs, Au NPs, and $MnFe_2O_4$ NPs), as well as through a bare silicon substrate, were measured by using terahertz time-domain spectroscopy (THz-TDS) in transmission modes. Under laser irradiation with different powers the THz amplitude transmissions through the four control samples (silicon coated with Ag NPs, Fe_3O_4 NPs, TiO_2 NPs and Au NPs), as well as for the bare silicon, slightly decreased with increasing laser power from zero to 1.4 W (shown in Figure S5). However, the THz amplitude transmission through silicon coated with $MnFe_2O_4$ NPs was sharply decreased with increasing laser power, whereby the transmission value

dropped to a minimum under the laser power of about 0.34 W. These experimental results demonstrated that within 5 different NP materials tested, the $MnFe_2O_4$ NPs had the best performance on enhancing the surface carrier concentration.

All the samples as investigated above contained metals (Ti, Ag, Au, Fe and Mn). The work function of metal materials on top of semiconductors can influence the band structure of the semiconductor at the interface between metal and semiconductor.[1-5] When the work function of a metal is lower than that of an adjacent semiconductor ($W_m < W_s$), many electron carriers are accumulated at the interface. The accumulated carriers induce an increased carrier density at the interface in the semiconductor, resulting in enhancement of the conductivity along the semiconductor surface.[1-5] In comparison with the work functions of the other investigated materials, the work function of Mn (W_{Mn}) is the lowest, as shown in Figure S6. In this case, the work function of Mn is lower than that of silicon ($W_{Mn} < W_{si}$). W_{Mn} is of influence for the work function of $MnFe_2O_4$. In addition, the work function of GaAs was described in reference.[6] When a material contacts a semiconductor, the work function of contacted material is one factor which affects the change of band structure of the semiconductor. However, according to some references,[1,2,4,5,7,8] the surface state of contacted material is another key factor affecting change of band structure of semiconductors. In the nano region, surface states of nanomaterials (such as NPs) have a significant effect on surface electrical properties of semiconductors. Surface states of nanoparticles originate from the surface atoms of NPs, which present weak and unsaturated bonding states, which drastically influence their surface properties. The different NPs have different surface states, which resulted in different effects on semiconductors. The surface state of $MnFe_2O_4$ NPs perhaps has a significant effect on the semiconductor substrate. In conclusion, $MnFe_2O_4$ NPs can provide more enhancement of the conductivity along the semiconductor surface as compared to the other NPs investigate here. Therefore, we chose $MnFe_2O_4$ NPs in this study for all detailed measurements.

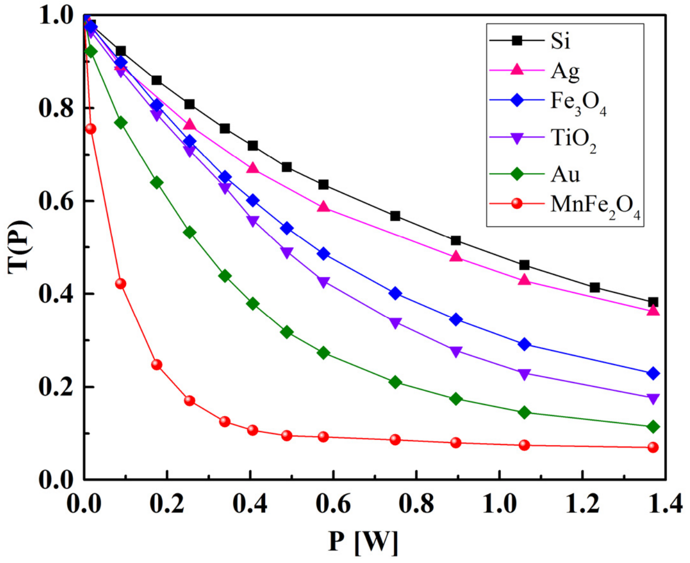

Figure S5. Amplitude transmissions of the terahertz pulses transmitted through the silicon coated with NPs and the bare silicon under laser irradiation with different powers P, respectively.

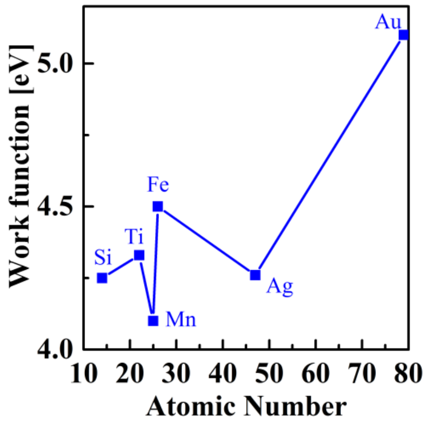

Figure S6. Work functions of materials [1-5]

Besides, in order to exclude the effect of magnetic response of NPs on the surface photoconductivity of the semiconductors, different composite magnetic nanoparticles (NPs) with similar size were compared, including $MnFe_2O_4$ NPs, Fe_3O_4 NPs and $CoFe_2O_4$ NPs. From Figure S7 we can see, all of them exhibit excellent monodispersity with uniform size around 15 nm, and the M-H curves showed superparamagnetic behaviour with similar smooth loops. The saturation magnetization (Ms) of $MnFe_2O_4$ NPs is a little bit higher than the

others; while, the THz transmission measurement based on $MnFe_2O_4$ NPs present significantly improvement compared to other two types of magnetic NPs, as shown in Figure S8. Therefore, the influence of magnetic response of nanoparticles on surface photoconductivity of the semiconductors seems negligible.

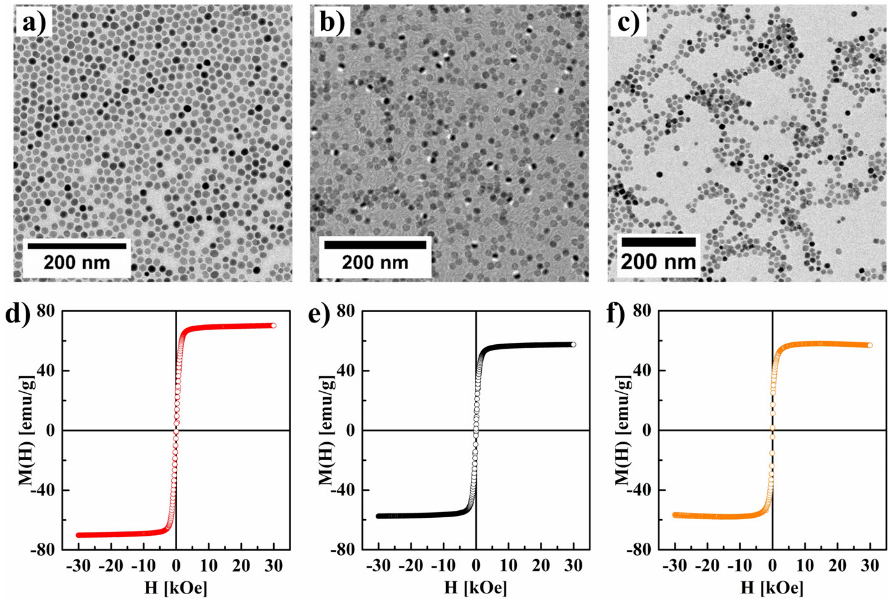

Figure S7. TEM images of three magnetic NPs. a) $MnFe_2O_4$ NPs; b) Fe_3O_4 NPs and c) $CoFe_2O_4$ NPs. All the samples were dispersed in chloroform before the actual TEM measurements carried out in vacuum. Magnetization versus magnetic field M(H) curve of three magnetic NPs, d) $MnFe_2O_4$ NPs; e) Fe_3O_4 NPs and f) $CoFe_2O_4$ NPs, measured at 300 K using a SQUID magnetometer.

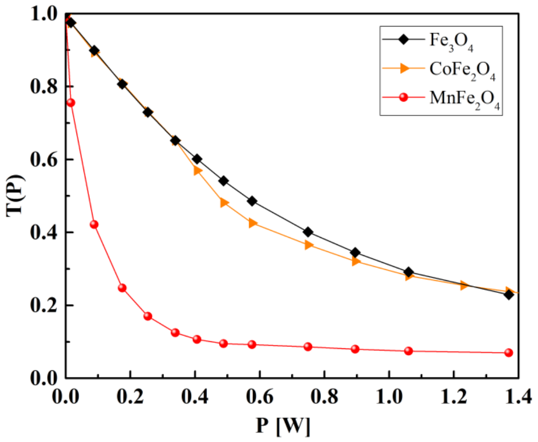

Figure S8. Amplitude transmissions of the terahertz pulses transmitted through the silicon coated with NPs ($MnFe_2O_4$ NPs, Fe_3O_4 NPs and $CoFe_2O_4$ NPs) under laser irradiation with different powers P, respectively.

5. Proposed theory: simulations based on band bending induced by $MnFe_2O_4$ NPs on silicon.

To account for the effect $MnFe_2O_4$ NPs on silicon in our observations, we propose the following model. When the silicon substrate is under laser irradiation, the volume of photo-excited carriers is defined by the irradiated area and the penetration depth of the laser beam. To account for the optical properties of the silicon under laser irradiation, the silicon can approximately be divided into a conducting layer and a lossless layer. Hereby, the thickness of the conducting layer is defined by the penetration depth of the laser beam, and the thickness of the lossless layer is defined by the part of the substrate with no laser irradiation. When the silicon is under laser irradiation, a carrier gradient exists between the surface and the interior part of the silicon, which can change the energy band structure of the interface between air and silicon. Therefore, photo-excited carriers in the silicon substrate diffuse from the surface into the interior to reach an equilibrium state.[1-5] Meanwhile, the photo-excited carriers induce a change of conductivity in the conducting layer of silicon. The optical system of NPOS substrates under laser irradiation can be approximately divided into a lossless layer of the silicon and a conducting layer consisting of the NPs and the photo-doped silicon layer. In the aforementioned experiment, the wavelength of the CW laser irradiating samples was 808 nm. According to the absorption spectrum of the $MnFe_2O_4$ NPs in Figure 1c, there was no apparent absorption of the $MnFe_2O_4$ NPs at 808 nm, so that the number of photo-excited carriers from the $MnFe_2O_4$ NPs was negligible. Therefore, the conductivity of NPOS substrates under laser irradiation is mainly dominated by the conductivity of the conducting layer in the silicon.[2,4,9] However, the surface atoms of the $MnFe_2O_4$ NPs present an unsaturated bonding state and have high chemical potential.[10-12] These surface atoms of the $MnFe_2O_4$ NPs change the energy band structure of the interface between the $MnFe_2O_4$ NPs and the silicon. The surface state of the $MnFe_2O_4$ NPs directly causes band bending of the interface between NPs and silicon. It also influences the quasi-Fermi level splitting in NPOS substrates under laser irradiation. Due to the effect of the surface states of the $MnFe_2O_4$ NPs, the diffusion of the photo-excited carriers in the silicon is more restricted to the surface, than in the case of substrates without NPs. The photo-excited carriers in silicon mainly accumulate at the interface between the $MnFe_2O_4$ NPs and silicon. The accumulated carriers induce an increased photo-carrier density on the interface between the $MnFe_2O_4$ NPs and silicon, resulting in enhancement of the conductivity on the silicon surface. In the

comparison with NPOS substrates, the photo-excited carriers of bare silicon substrates mostly diffuse from the surface into the interior because of the existence of a carrier gradient between surface and interior in the bare silicon. Therefore, under laser irradiation with the same power, the conductivity of the conducting layer in the bare silicon is much lower than that of the conducting layer in NPOS substrates. Actually, the conducting layer in the bare silicon is much thicker than that in NPOS substrates. However, photo-excited carriers of the bare silicon mostly diffuse from the surface into the interior, and a part of photo-excited carriers decay in the diffusion process. Hence, the part of the conducting layer in the bare silicon, which exceeds the thickness of the conducting layer in NPOS substrates, has low conductivity and could be almost negligible. Therefore, the thickness of the conducting layer of the NPOS substrate and the bare silicon could be approximately same in the simulations.

In the model for NPOS substrates under laser irradiation, the electrical properties of silicon can be described by holes in the valence band and electrons in the conduction band.[2,5] Concentrations of holes and electrons are dominated by the quasi-Fermi levels of holes and electrons in the quasi-thermal equilibrium. In terms of the quasi-Fermi levels of holes and electrons, the conductivity σ in silicon can be written as:

$$\sigma = e \cdot (\mu_n + \mu_p) \cdot n_i \cdot e^{\frac{\Delta E_{n,h}}{2 \cdot K_B T}}$$

Where e is the electron charge, μ_n is the mobility of electrons, μ_p is the mobility of holes, n_i is intrinsic carrier concentration of silicon, $\Delta E_{n,h}$ is the separation energy ($\Delta E_{n,h} = E_{Fn} - E_{Fh}$) between the electron quasi-Fermi level E_{Fn} and hole the quasi-Fermi level E_{Fh}, K_B is the Boltzmann constant, and T is the Kelvin temperature, respectively.

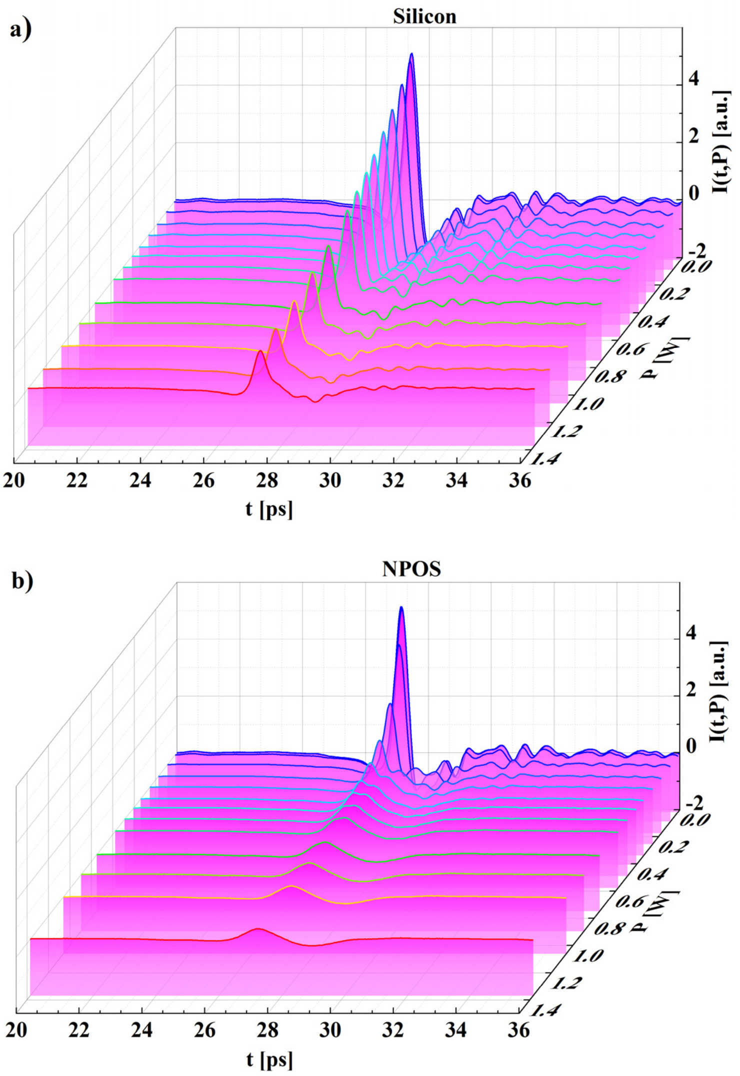

Figure S9. Waveforms of the THz pulses transmitted through the silicon and NPOS under the laser irradiation with different powers.

6. Numerical Methods

The simulations of the proposed model were performed by electromagnetic simulation software. We chose the thickness of the conducting layer of the silicon defined by the penetration depth of the laser beam, which was about 5 μm. The thickness of the lossless layer of silicon was 510 μm. In that way, the total thickness of the silicon used in the experiment consists of the thicknesses of the conducting layer and the lossless layer of silicon. By this means, we determined the silicon conductivity with dependence on the laser beam power. The

parameters of silicon are from the literature values: $\mu_n = 1400\ \text{cm}^2/(\text{V}\cdot\text{s})$, $\mu_p = 450\ \text{cm}^2/(\text{V}\cdot\text{s})$, $N_c = 3.22\times10^{19}\ \text{cm}^{-3}$, $N_v = 1.83\times10^{19}\ \text{cm}^{-3}$ and $E_g = 1.12\ \text{eV}$ for silicon at room temperature.

References

1 Neamen, D. *Semiconductor Physics And Devices*. (McGraw-Hill, Inc., 2002).

2 Kittel, C. *Introduction to solid state physics*. (Wiley, 2004).

3 Young, C. D. Exploring terahertz pulse enhancement through gold nanoparticle deposition. *Dissertations & Theses - Gradworks* (2009).

4 Fox, M. *Optical properties of solids*. Vol. 3 (Oxford University Press, 2010).

5 Sze, S. M. *Semiconductor devices: physics and technology*. (John Wiley & Sons, 2008).

6 Haneman, D. Photoelectric emission and work functions of InSb, GaAs, Bi_2Te_3 and germanium. *Journal of Physics & Chemistry of Solids* **11**, 205,IN201,209-208,IN202,214 (1959).

7 Grundmann, M. *The physics of semiconductors:an introduction including Nanophysics and applications*. (Springer, 2014).

8 Sze, S. M. & Mattis, D. C. *Physics of Semiconductor Devices*. (Wiley-Interscience, 2007).

9 Weis, P. *et al.* Spectrally Wide-Band Terahertz Wave Modulator Based on Optically Tuned Graphene. *Acs Nano* **6**, 9118-9124 (2012).

10 Antonov, V. N., Harmon, B. N. & Yaresko, A. N. Electronic structure and x-ray magnetic circular dichroism in Fe3O4 and Mn-, Co-, or Ni-substituted Fe3O4. *Phys Rev B* **67** (2003).

11 Bateer, B. *et al.* Synthesis, size and magnetic properties of controllable MnFe2O4 nanoparticles with versatile surface functionalities. *Dalton T* **43**, 9885-9891 (2014).

12 Singh, D. J., Gupta, M. & Gupta, R. First-principles investigation of MnFe2O4. *Phys Rev B* **65** (2002).
